# *Scipion* Flexibility Hub: an integrative framework for advanced analysis of conformational heterogeneity in cryoEM

**DOI:** 10.1107/S2059798323004497

**Published:** 2023-06-16

**Authors:** D. Herreros, J. M. Krieger, Y. Fonseca, P. Conesa, M. Harastani, R. Vuillemot, I. Hamitouche, R. Serrano Gutiérrez, M. Gragera, R. Melero, S. Jonic, J. M. Carazo, C. O. S. Sorzano

**Affiliations:** a Centro Nacional de Biotecnologia – CSIC, Calle Darwin 3, 28049 Madrid, Spain; b IMPMC–UMR 7590 CNRS, Sorbonne Université, MNHN, Paris, France; c Escuela Politécnica Superior, Calle Francisco Tomás y Valiente 11, 28049 Madrid, Spain; Rutherford Appleton Laboratory, United Kingdom

**Keywords:** single-particle analysis, image processing, cryo-electron microscopy, protein dynamics, continuous conformational variability, *Scipion* Flexibility Hub

## Abstract

Understanding how structure and function meet to drive biological processes is progressively shifting the cryoEM field towards a more advanced analysis of macromolecular flexibility. Thanks to techniques such as single-particle analysis and electron tomography, it is possible to image a macromolecule in different states, information that can subsequently be extracted through advanced image-processing methods to build a richer approximation of a conformational landscape. However, the interoperability of all of these algorithms remains a challenging task that is left to users, preventing them from defining a single flexible workflow in which conformational information can be addressed by different algorithms. Therefore, in this work, a new framework integrated into *Scipion* is proposed called the Flexibility Hub. This framework automatically handles intercommunication between different heterogeneity software, simplifying the task of combining the software into workflows in which the quality and the amount of information extracted from flexibility analysis is maximized.

## Abstract 2

An integrative framework called Flexibility Hub is integrated into *Scipion* that simplifies the interoperability of different heterogeneity algorithms.

## 1. Introduction

CryoEM single-particle analysis (SPA; Carroni & Saibil, 2016 ▸) is currently one of the most successful techniques in structural biology, with the ability to resolve high-resolution structures of large macromolecules. By capturing macromolecular particles in different poses, it is possible to reconstruct high-resolution maps by estimation of the projection geometry for each imaged particle.

However, due to the dynamic nature of macromolecules, SPA does not capture macromolecules in a single stable conformation. Each imaged particle might represent a different conformational state, so that the ensemble of all of them provides very rich information about the conformational landscapes representing the different transitions and states explored by the macromolecule (at least within experimental limitations mainly related to image-acquisition issues).

The possibility of extracting dynamic structural information from cryoEM data sets is inducing a shift in the field by the development of advanced algorithms to analyse molecular flexibility from cryoEM data. However, all of these new algorithms have been independently implemented in separate programs and they are not easy to apply and combine or compare.

**Figure 1:**
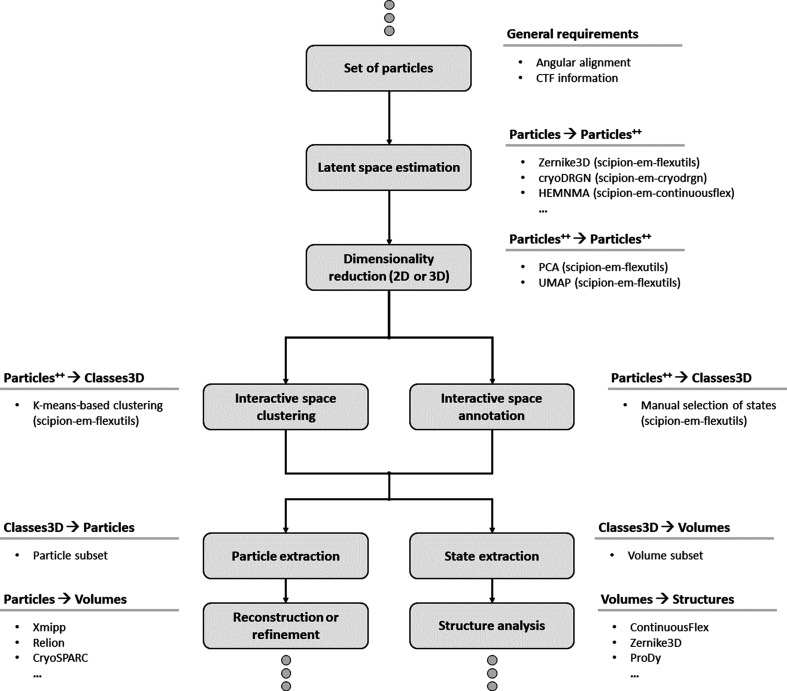
Simplified scheme of a Flexibility Hub workflow. For simplicity, the example only shows how to start from a set of particles, although working with volumes or structures is also possible. The top ellipsis is used to indicate that the input particles in the workflow might be obtained by different means (imports, refinements *etc.*) and the bottom ellipses show that the output of the flexibility workflow might also be connected to other workflows, protocols or plugins inside *Scipion*. The label ‘Particles^++^’ refers to a set of particles that has been extended with new features (*Zernike*3*D* coefficients, *cryoDRGN* latent space vectors, PCA or UMAP features *etc.*).

To this end, we have integrated many of the newest tools into *Scipion* (Rosa-Trevín *et al.*, 2016 ▸) in a new centralized framework called the Flexibility Hub. The main purpose of the new framework is to provide users with a simple and intuitive way to apply and combine the currently integrated flexibility tools to increase the knowledge of the dynamics and heterogeneity that can be drawn from a data set. An example of a Flexibility Hub workflow template is provided in Fig. 1 ▸, which exemplifies an execution flow that could be designed inside *Scipion* to analyse the heterogeneity of a data set.

Before we present the details of our implementation and some examples of the workflows that it enables, we first provide a brief overview of the different kinds of flexibility tools that we have considered. The general goal in the type of cryoEM analyses we address in this work is to have some form of description of the specific conformational changes exposed by a certain data set that is being analysed without any explicit restriction to these structural changes being discrete and well represented by a certain number of ‘classes’; note that different methods use different mathematical approaches, including different representations for each particle image. Depending on the method used, the mathematical approach might describe the mapping of a particle state to a volume, allowing heterogeneous reconstructions of the particle states to be performed. On the other hand, some algorithms estimate a deformation field, which can be applied to a common reference state (map or atomic structure) to approximate the conformational state represented by a given particle. The goal is then to analyse the ensemble of all of these changes in a way that allows an estimation of which of them are more common (energetically favourable), how they relate to each other and the possible existence of trajectories between the different conformations.

Some continuous heterogeneity algorithms explicitly try to estimate the deformation field relating each particle to some reference conformation, such as *Zernike*3*D* (Herreros *et al.*, 2021 ▸, 2023 ▸), *TomoFlow* (Harastani, Eltsov *et al.*, 2022 ▸) and *HEMNMA* (Jin *et al.*, 2014 ▸). In contrast, other algorithms such as *cryoDRGN* (Zhong *et al.*, 2021 ▸), *GMM* (Chen & Ludtke, 2021 ▸) and *ManiFoldEM* (Frank & Ourmazd, 2016 ▸) directly estimate the deformed map without an explicit estimation of the underlying deformation field.

**Figure 2:**
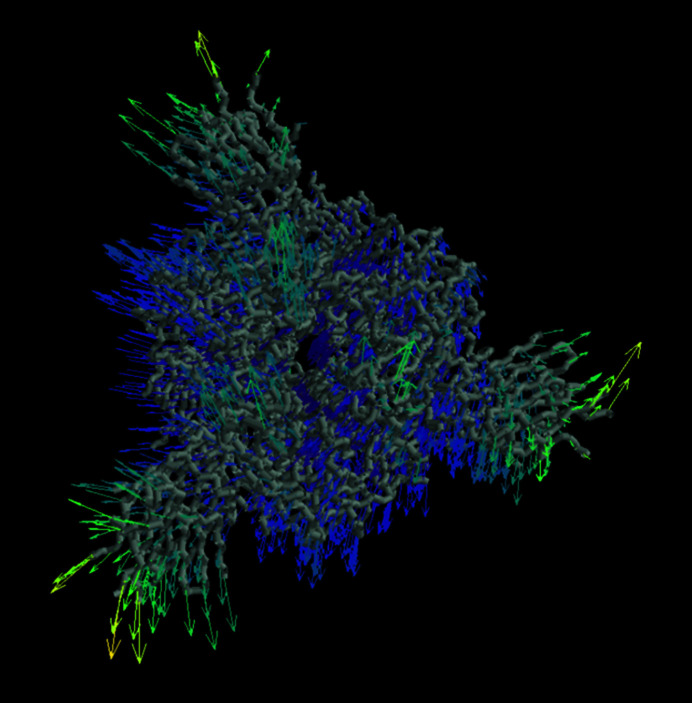
Example of an interactive visualization of the local motion statistics computed for the 3Down spike from the deformation fields estimated using the *Zernike*3*D* algorithm. The arrows represent the average direction of motion of the spike C^α^ atoms. The arrows are coloured according to the standard deviation of the motion of each atom according to the deformation fields.

Each approach has its own advantages and disadvantages. For example, explicitly estimating the deformation field allows the visualization of local characteristics of the motions associated with each atom in the macromolecule, such as the deformation field describing a given conformational transition that is provided in Fig. 2 ▸, as well as calculation of the local strains and rotations experienced by a macromolecule that is undergoing such deformations (Sorzano *et al.*, 2016 ▸). Methods that directly estimate the maps, on the other hand, can more easily account for compositional heterogeneity, aiding in understanding the structural information that is present in different groups of particles.

## 2. Methods

The Flexibility Hub has been designed as a new section inside *Scipion* to wrap algorithms related to the estimation, inspection, analysis or understanding of conformational landscapes captured in the different cryoEM data types (particles, maps and structural models) by several different flexibility analysis methods.

The core of the Flexibility Hub is the new *Flexutils* plugin, which contains the main visualization and analysis tools required by the other plugins to connect their outputs into a meaningful workflow. However, it is still possible to use some of these plugins on their own (such as *ContinuousFlex*) as they include their own analysis tools inside their plugin and thus do not need to rely completely on *Flexutils*.

In addition, *Flexutils* also contains all of the *Zernike*3*D*- and *ZART*-related programs (Herreros *et al.*, 2023 ▸), which themselves provide users with tools to address the flexibility of their data. All the other software (*cryoDRGN*, *ContinuousFlex* *etc.*) can be installed either through the *Scipion Plugin Manager* (automatic installation) or through their corresponding GitHub pages (*scipion-em-cryodrgn*, *scipion-em-continuousflex* *etc.*; manual installation).

In Fig. 1 ▸ we provide a heterogeneity workflow template with some possible steps to analyse the heterogeneity of a set of particles. The steps are restricted to the case of estimating landscapes from particles only. However, more advanced workflows could be defined, and other data types could also be used or added to the workflow.

It is also worth mentioning that the Flexibility Hub should not be considered an isolated section of *Scipion*; it is possible to connect the heterogeneity workflows to any other standard SPA plugin or protocol. For example, it is possible to use the tools inside *CryoSPARC* (Punjani *et al.*, 2017 ▸) or *RELION* (Scheres, 2012 ▸) plugins to reconstruct different regions of a conformational landscape from a set of selected particles.

### 2.1. Methods that explicitly estimate the deformation field

In this section, we describe the various methods implemented in *Scipion* for continuous heterogeneity analysis by explicitly estimating the underlying deformation field. In particular, we provide *Zernike*3*D* (which estimates a continuous description of the deformation field), *HEMNMA* and related methods (which allow estimation and analysis of the deformation field based on its samples at the positions of the macromolecule atoms or pseudoatoms using normal modes), *TomoFlow* (which estimates the deformation field from sub­tomograms) and *NMMD* (which estimates the deformation field incorporating knowledge from both normal modes and molecular-dynamics simulations). As all of these methods try to estimate the deformation field, they can be interconverted. For instance, the normal mode amplitudes can be estimated from the *Zernike*3*D* amplitudes, or vice versa, as described below.

#### 2.1.1. *Zernike*3*D*

The *Zernike*3*D* method (Herreros *et al.*, 2021 ▸) is a new approach to studying continuous flexibility at different levels depending on the available data. The approach is based on the estimation of a deformation field that describes the motion that a molecule has experienced to reach a different state. One of the main advantages of the *Zernike*3*D* approach is the possibility of applying it indistinguishably to cryoEM maps, particle images and structural models, translating them to a common space. This opens the door to integrative heterogeneous analysis from different studies or sources.

The *Zernike*3*D* workflow has been fully integrated into *Scipion*, providing advanced and novel protocols for the analysis of flexible specimens. The various steps of the workflow are summarized in the next sections.

**Zernike*3*D* coefficient estimation*. The programs in charge of estimating the *Zernike*3*D* coefficients α_*l*,*n*,*m*_ for structural models, maps and particles have been protocolized in *Scipion*, providing the initial step of the* Zernike*3*D* workflow. The protocols allow the estimation and visualization of approximate conformational landscapes estimated from data of these various types to perform an initial assessment of the different conformational changes represented by the data.

An example of the *Scipion* input form of the *Zernike*3*D* coefficient-estimation protocol for particles is provided in Supplementary Fig. S1(*a* ).

The form of this protocol offers the required parameters interactively based on the information stored in the provided input particles. Input particles must have angular alignment information registered as well as CTF information. However, if the particles have been CTF-corrected it is possible to use the advanced parameters in the form to execute the analysis without the need for CTF information.

In addition, depending on the information stored in the particles, some other parameters will also be offered, such as the reference map to be used for the analysis.

The output of this protocol will be registered as a *Scipion* set of particles extended with the *Zernike*3*D* coefficients estimated for each particle.

*Motion-corrected reconstruction with ZART*. The *Zernike*3*D* workflow includes a novel reconstruction algorithm called *ZART* (*Zernike3D-based Algebraic Reconstruction Technique*; Herreros *et al.*, 2023 ▸). This new algorithm uses the estimated deformation fields to correct the molecular motions during the reconstruction process, thus increasing the local resolution of the map regions affected by motion-blur artefacts.

This new reconstruction approach is also included in the *Scipion* *Zernike*3*D* workflow, including all of the reconstruction modes provided by the method (standard reconstruction, gold standard and multi-resolution).

An example of the *ZART* protocol input form is provided in Supplementary Fig. S1(*b*). In the case of *ZART*, input particles are the only mandatory input, and should have angular alignment information and CTF (in the case where the particles are not CTF-corrected). Heterogeneity correction also requires that the input particles have been generated by a *Zernike*3*D* coefficient-estimation protocol.

A *ZART* protocol will register the reconstructed map as output in *Scipion*, which might include half maps if the gold-standard or multi-resolution modes have been selected.

**Figure 3:**
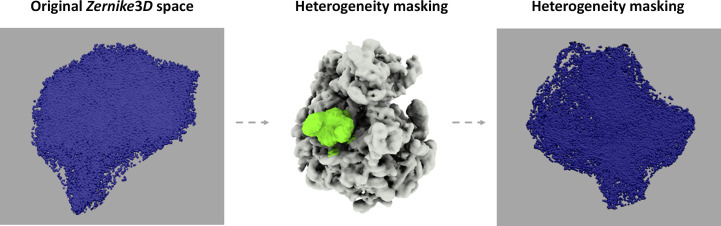
Description of the focused heterogeneity workflow applied to the EMPIAR-10028 data set. The analysis reveals a new conformational landscape describing in a more informative manner the conformational changes affecting the L1 stalk region, which would remain hidden in the original landscape due to the size difference between the region of interest and the whole ribosome. In this way, it is possible to more easily inspect the localized conformational changes affecting the selected region in order to increase the knowledge of localized molecular motions.

*Focused heterogeneity analysis*. Another step implemented in *Scipion* consists of a series of tools to focus a conformational landscape on a given region of a macromolecule. In this way, it is possible to study only the conformational changes belonging to a given region of interest, which might remain hidden if the whole molecule is considered. An example of a focused heterogeneity landscape is provided in Fig. 3 ▸, and Supplementary Video S1 shows an example of the *k*-means exploration of the original and focused landscapes.

The focused heterogeneity analysis form is shown in Supplementary Fig. S1(*c*). The required parameters include a set of particles registered by a *Zernike*3*D* coefficient-estimation protocol and a mask delimiting the region in which the landscape is to be focused.

The protocol will register a new set of particles with updated *Zernike*3*D* coefficients corresponding to the focused landscape.

*Reference reassignment and *ZART**. The *Zernike*3*D* algorithm relies on the information of a reference map to compute and approximate the deformation fields describing a given molecular motion. Therefore, the deformation fields can only be applied to the reference map provided as input to the *Zernike*3*D* method.

**Figure 4:**
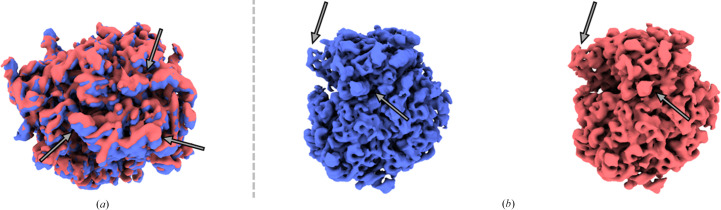
Example of two *ZART* reconstructions representing the two main conformational states in the EMPIAR-10028 data set. The two states correspond to the conformations present in the data set, corresponding to a movement of the small subunit of the ribosome. Thanks to *ZART*, it is possible to reconstruct these two states without sacrificing any particles, thus increasing the resolution of the resulting cryoEM maps. (*a*) Comparison of the two states reconstructed by *ZART*. The comparison shows the motion of the small subunit of the ribosome. Arrows are added to aid the visualization of the conformational change. (*b*) Visualization of the two reconstructed maps. As expected, both maps show similar features, even though one of the reconstructed states has a much lower representation in the landscape.

This implies that the flexibility-corrected maps reconstructed by *ZART* will always be in the same conformation as the reference. However, we have included in *Scipion* an approach to reassign the *Zernike*3*D* deformation fields to any other conformation represented in the estimated conformational landscape, as exemplified in Fig. 4 ▸ and Supplementary Video S2. Therefore, combination of the reference-assignment algorithm and *ZART* allows the reconstruction of any conformation of the landscape without losing any particle in the process, leading to high-resolution structures even if a conformation is not well represented.

The reassignment of the deformation fields to a different conformation relies on the possibility of computing new deformation fields through the subtraction and addition of the currently estimated fields. We can express the deformation field of conformation *X* associated with a reference volume *R* as

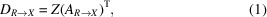

where *Z* is the *Zernike*3*D* basis computed at the valid positions of the reference *R* (such as only those locations within the mask if one was provided) and *A*_*R*→*X*_ are the *Zernike*3*D* deformation coefficients needed to move the reference towards conformation *X*. The transpose of the *Zernike*3*D* coefficients is added to match the notation in Section 2.1.4.

In addition, the reassignment also needs to have the deformation field that drives the current reference map towards the new reference *R*′, which can be expressed as 

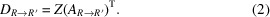




From the two previous results, it is possible to obtain the deformation field to be applied to *R*′ to reach conformation *X* as 

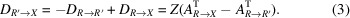




Moreover, it is possible to calculate the *Zernike*3*D* coefficients for the new reference (further information on the derivation is given in Section 2.1.4),

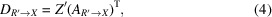




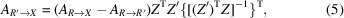

where *Z*′ is the *Zernike*3*D* basis evaluated on the new reference map *R*′ and 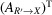 is the set of *Zernike*3*D* coefficients estimated for the new reference. It must be noted that *Z* and *Z*′ may not be the same, as the masks for the *R* and *R*′ maps may be different. If no masks are provided for either of the two maps then *Z* = *Z*′ and the expression above simplifies to 

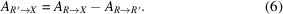




The parameters to be filled in the form of the reference-reassignment protocol are shown in Supplementary Fig. S1(*d*). The required parameters include a set of particles estimated with a *Zernike*3*D* coefficient-estimation protocol, a mask associated with the reference volume used to estimate the *Zernike*3*D* coefficients of the input particles and a new mask delimiting the region in which the new reference is found.

The protocol will register a new set of particles whose *Zernike*3*D* coefficients have been reassigned to the new specified reference.

#### 2.1.2. ContinuousFlex

*ContinuousFlex* is a software package that allows analysis of the continuous conformational variability of biomolecular complexes in cryoEM single-particle images and cryo-electron tomography (cryoET) sub­tomogram data by combining data analysis and molecular-dynamics prediction based on simulations or deep learning. *ContinuousFlex* is available as a plugin for *Scipion*.

The tools currently available in *ContinuousFlex* allow the data-analysis methods published under the names *HEMNMA* (Jin *et al.*, 2014 ▸; Harastani *et al.*, 2020 ▸), *DeepHEMNMA* (Hamitouche & Jonić, 2022 ▸), *HEMNMA*-3*D* (Harastani *et al.*, 2021 ▸), *TomoFlow* (Harastani, Eltsov *et al.*, 2022 ▸), *NMMD* (Vuillemot *et al.*, 2022 ▸), *MDSPACE* (Vuillemot, Mirzaei *et al.*, 2023 ▸) and *MDTOMO* (Vuillemot, Rouiller *et al.*, 2023 ▸) to be run. Each of these methods has its own workflow, which is fully described in a recently published review article on *ContinuousFlex* (Harastani, Vuillemot *et al.*, 2022 ▸). They are briefly reviewed in this section.

*ContinuousFlex* is under continuous development. New methods and new features of the currently available methods will be available in *ContinuousFlex* in the future.

*HEMNMA.* *HEMNMA* allows analysis of the conformational variability in a given set of single-particle images using normal modes of an initial conformation determined from an input atomic structure or cryoEM map (Jin *et al.*, 2014 ▸; Harastani *et al.*, 2020 ▸). When the initial conformation is given by a cryoEM map, this map is converted into 3D Gaussian functions (referred to as pseudoatoms; Jonić & Sorzano, 2016 ▸) and the coordinates of their centres serve as the atomic coordinates for normal mode analysis (NMA).

NMA of the initial conformation is performed using an elastic network model (ENM; Tirion, 1996 ▸), as implemented in *ElNémo* (Suhre & Sanejouand, 2004 ▸), which is available in *ContinuousFlex*. NMA approximates the complex motion as a linear combination of simple harmonic oscillator-type motions of different frequencies. These are called normal modes and are vectors along which atoms or pseudoatoms move. Analysis of each particle image by *HEMNMA* produces an estimate of the corresponding amplitudes of motion along each normal mode used, which determine the conformation of the particle in this image.

*HEMNMA* simultaneously determines the parameters of conformation (normal-mode amplitudes) and of the rigid-body pose (three Euler angles and two shifts) of the particle in each image by 3D-to-2D elastic (using normal modes) and rigid-body fitting of the initial conformation to the image through parallel (MPI-based) processing of the images. The conformational parameters obtained for all images are mapped onto a low-dimensional space (by principal component analysis or another dimension-reduction technique), which describes an essential conformational space or conformational landscape.

Animations of the conformational transitions of the given input model can be obtained along different directions through dense regions of this space. Additionally, 3D reconstructions can be obtained by grouping images with similar conformations (corresponding to close points) in this space.

*DeepHEMNMA*. *DeepHEMNMA* employs a deep neural network to speed up the processing of large data sets by *HEMNMA* (Hamitouche & Jonić, 2022 ▸). The network is first trained to learn the relationships between a small subset of single-particle images and their corresponding conformational and pose parameters obtained with *HEMNMA*. The trained network is then used to infer these parameters for the remaining images. *DeepHEMNMA* has been shown to be at least 40 times faster than *HEMNMA* (Hamitouche & Jonić, 2022 ▸).

*HEMNMA-3D*. *HEMNMA*-3*D* is an extension of *HEMNMA* to conformational variability analysis in sub­tomogram data (Harastani *et al.*, 2021 ▸). The conformational and pose parameters of the particle in each subtomogram are determined by 3D-to-3D elastic (using normal modes) and rigid-body fitting of the initial conformation (given by an atomic structure, a cryoEM map or a subtomogram average computed from the given set of subtomograms) to the sub­tomogram through parallel (MPI-based) processing of the given set of subtomograms. As in *HEMNMA*, the elastic conformational parameters are normal-mode amplitudes. The rigid-body pose parameters are three Euler angles and three shifts. The low-dimensional conformational space obtained with *HEMNMA*-3*D* can be analysed in terms of animations of conformational changes (as with *HEMNMA*) or in terms of subtomogram averages calculated from subtomograms with similar conformations (corresponding to close points in space).

Supplementary Fig. S2 shows some of the protocols that could be defined to design a *HEMNMA* workflow. The workflow is subdivided into three different steps: extraction of a pseudoatomic structure from a cryoEM map (Supplementary Fig. S2*a* ), computation of normal modes from an atomic or pseudoatomic structure (Supplementary Fig. S2*b* ) and assignment of normal mode coefficients to a set of particles based on a set of previously computed normal modes (Supplementary Fig. S2*c* ).

*TomoFlow*. *TomoFlow* allows conformational variability analysis in subtomogram data using the computer vision technique of optical flow (Harastani, Eltsov *et al.*, 2022 ▸). Optical flow determines the displacement of each voxel between two given density maps. *TomoFlow* uses optical flow for a combined elastic and rigid-body alignment between each given subtomogram and the density map of the initial conformation (an input cryoEM map, an atomic structure converted into a density map or a subtomogram average computed from the given set of subtomograms). The optical flows calculated between rigid-body aligned subtomograms and the initial conformation density map are mapped onto a low-dimensional space, which represents the essential conformational landscape, which can be analysed as in the case of the conformational landscape obtained with *HEMNMA*-3*D*. An advantage of *TomoFlow* is that it extracts information about the conformational variability from the data without requiring any prior information about the dynamics of the given molecular system, in contrast to *HEMNMA*-3*D* which requires information about the potential motion directions (normal mode vectors). A disadvantage of *TomoFlow* is that the currently used optical flow implementation involves a large amount of data smoothing, which may reduce the amplitude of the motion extracted from the data. *HEMNMA*-3*D* and *TomoFlow* were used to analyse *in situ* nucleosome subtomograms and both revealed nucleosome motions that had previously only been shown *in silico* and *in vitro* (nucleosome breathing and gapping; Harastani, Eltsov *et al.*, 2022 ▸).

*NMMD*. *NMMD* is a method for 3D-to-3D flexible fitting of an atomic structure to a cryoEM map, which efficiently combines the atomic displacement using classical, force-field molecular-dynamics (MD) simulation with a displacement along normal modes (Vuillemot *et al.*, 2022 ▸). MD simulation is performed using *GENESIS* version 1.4 (Kobayashi *et al.*, 2017 ▸), which is available in *ContinuousFlex*. *ContinuousFlex* allows the use of the following force fields (provided by *GENESIS*): CHARMM (Huang & MacKerell, 2013 ▸) and Go model (all-atom or C^α^-atom based; Karanicolas & Brooks, 2003 ▸). *NMMD* was implemented by adding normal modes to the flexible fitting method based solely on MD simulation (Orzechowski & Tama, 2008 ▸), which was already available in *GENESIS* and is now also available in *ContinuousFlex*. The fitting method that uses a combination of normal modes and MD simulation (*NMMD*) showed a better performance than the method using MD simulation only (Vuillemot *et al.*, 2022 ▸). *NMMD* more efficiently fits both large-scale motions (thanks to the use of normal modes) and finer motions (thanks to MD simulation) (Vuillemot *et al.*, 2022 ▸).

*MDSPACE* and *MDTOMO* are two approaches that use *NMMD* to analyze biomolecular conformational dynamics in single particle images (Vuillemot, Mirzaei *et al.*, 2023 ▸) and subtomograms (Vuillemot, Rouiller *et al.*, 2023 ▸), respectively. They produce conformational variability landscapes at atomic scale while successfully describing both global and local conformational dynamics.

#### 2.1.3. ProDy

*ProDy* (Zhang, Krieger, Zhang *et al.*, 2021 ▸) is a Python application programming interface (API) for protein dynamics studies including ENM NMA and ensemble analysis of structural models using principal component analysis (PCA), which provides a wide range of tools as a library for proficient programmers. In order to make these methods more available to structural biologists, we integrated them into a *Scipion* plugin including a viewer connected to the normal mode wizard, *NMWiz*, in *VMD*. This plugin provides three main workflows.

*Building ensembles of structural models and ensemble analysis via PCA*. An ensemble analysis at the level of structural models can be very helpful in calculating the key motions available to a system. A very common way to perform this is via PCA using the positional covariance matrix of corresponding atoms after superposition to the average, yielding components of conformational variation similar to normal modes. The protocols related to this analysis are shown in Supplementary Figs. S3(*a*)–S3(*c* ).

*ProDy* contains various tools for aligning structural models into ensembles of corresponding atoms as well as tools for retrieving related structures from the PDB (Zhang *et al.*, 2019 ▸; Zhang, Krieger, Zhang *et al.*, 2021 ▸). The ensemble-building protocol in the plugin can either take individual structural models and sets of structural models from *Scipion* or retrieve them from the PDB with the *ProDy* interface to the *DALI* web server (Holm, 2022 ▸). In the case of a set of structural models, corresponding residues can be identified either by pairwise sequence alignment from Biopython or by combinatorial extension structural alignment. In the case of *DALI*, the mappings from *DALI* are used instead. The outputs are currently a set of aligned structural models with corresponding atoms.

The form of this protocol is shown in Supplementary Fig. S3(*a*). It also includes an atom-selection option and a chain-matching algorithm to optimize the r.m.s.d. and sequence-alignment quality, as well as an option to use matching chain IDs or orders. In our case, in which all of the structures from *Zernike*3*D* had the same atoms, this protocol was just used to select C^α^ atoms and perform iterative superposition.

There are then protocols for PCA and the projection of ensembles onto PCs or normal modes for analysis of conformational landscapes. The PCA protocol (Supplementary Fig. S3*b* ) takes the output of the ensemble-building protocol as its input and produces a set of principal components, which it inherits from the set of normal mode objects used by *ContinuousFlex* and therefore has access to the same viewers and protocols.

The projection protocol (Supplementary Fig. S3*c* ) takes the outputs from both ensemble construction and PCA as inputs and registers projection coefficients in *Scipion* that can be visualized with an associated viewer, creating a bar graph or by-conformer line graph for a single component or a 2D or 3D scatter plot for two or three components.

*Anisotropic network model normal mode analysis and deformation vectors*. The anisotropic network model (ANM; Atilgan *et al.*, 2001 ▸; Eyal *et al.*, 2006 ▸) is a simple elastic network model with uniform springs that is optimized for C^α^ atoms by default, allowing faster and more versatile normal mode analysis. For example, it is connected to various frameworks, such as vibrational subsystem analysis (VSA; also known as model reduction) for incorporating the effects of environmental parts of the structure such as membrane or flexible loops on the dynamics of the remaining subsystem (Hinsen *et al.*, 2000 ▸; Ming & Wall, 2005 ▸; Zheng & Brooks, 2005 ▸; Woodcock *et al.*, 2008 ▸).

In addition to the core protocol for ANM NMA (Supplementary Fig. S3*d* ), the *ProDy* plugin provides a protocol for atom selection with all the functionality of the *ProDy* API, including the selection of particular chains, residues and atom types, as well as a protocol for mode editing by reduction, slicing and extension using these selections. The output set of normal mode objects is the same as used in *ContinuousFlex*, allowing integration with its viewers and protocols such as *HEMNMA*. The inputs are atomic structure objects (which can be selected from sets as performed here) or PDB codes.

*ProDy* also provides tools for aligning pairs of structural models, including both sequence matching and superposition. Following these operations using these tools or using the ensemble tools above, the deformation vector between the two aligned structures can be calculated as the difference in the positions of the corresponding atoms. This allows the set of normal modes facilitating the transition to be evaluated following an appropriate mode-editing operation, such as slicing or reduction.

The projections of the deformation vector onto the mode vectors are calculated as dot products, providing ‘overlap’ or contribution coefficients that can be used to approximate the transition with the normal modes, which can be calculated using the ‘compare’ protocol. Typically, a small number (for example 20) of the lowest frequency, global modes are required in such an analysis to give a cumulative overlap (the square root of the sum of squared normalized overlaps) of around 0.8, accounting for the majority of the transition.

A similar calculation can also be used to assess the agreement between two sets of normal modes, for example between those calculated by *ProDy* and *ElNémo*/*ContinuousFlex*, between those calculated using different parameters or between normal modes and principal components, as performed here. These calculations also give an indication of which of these modes may be useful (robust and/or relevant) for downstream analyses such as *HEMNMA*.

*GNM analysis and dynamical domain decomposition*. Another analysis that can be performed with *ProDy* and its plugins is Gaussian network model (GNM) analysis (Bahar *et al.*, 1997 ▸). The GNM is an elastic network model based on the statistical mechanics of polymer networks under the assumption that the nodes and springs undergo Gaussian and isotropic fluctuations. It provides more accurate mechanical properties, including mean-square fluctuations and cross-correlations, and the identification of key regions such as hinges with minimal motion and dynamical domains that move together.

The analysis is similar to NMA and also relies on the decomposition of a connectivity matrix into modes describing the dynamics of a system, but these modes are *N*-dimensional rather than 3*N*-dimensional for a system of *N* nodes as a result of decomposing the Kirchhoff matrix and not the Hessian matrix. The pseudo-inverse of the Kirchhoff matrix from combining back the *N* − 1 nonzero modes gives rise to an *N* × *N* covariance matrix, which can be used to analyse mean-square fluctuations (MSFs) and cross-correlations.

The GNM analysis is provided as a protocol with a viewer for analysing mode shapes, MSFs and raw and normalized/orientational cross-correlations for individual modes and their combinations. There is also a protocol for dynamical domain decomposition related to spectral clustering (Zhang, Krieger, Mikulska-Ruminska *et al.*, 2021 ▸; Sauerwald *et al.*, 2017 ▸), which could be helpful in focused flexibility analyses such as those using *Zernike*3*D* (see Section 2.1.1) or *RELION* multi-body refinement (Nakane *et al.*, 2018 ▸).

#### 2.1.4. NMA–*Zernike*3*D* conversion

One of the main objectives of *Scipion* is the interoperability of different algorithms. Thanks to the abstraction level provided by *Scipion*, it is possible to reuse the information or parameters estimated among different algorithms in order to obtain the best possible results. Apart from the general interoperability tools available in *Scipion*, the Flexibility Hub framework enables new tools and conversion methods to move from NMA space to *Zernike*3*D* space (or, more specifically, to move that part of conformational space explained by NMA) and vice versa. In this way, it becomes possible to combine the *Zernike*3*D*, *ContinuousFlex* and *ProDy* protocols into more advanced workflows to study molecular flexibility.

The following two sections provide a detailed explanation of how to achieve these conversions.

*Conversion of *Zernike*3*D* space to NMA space*. The NMA–*Zernike*3*D* conversion is based on a least-squares approximation that tries to find the NMA or *Zernike*3*D* space that accurately reproduces a given deformation field.

According to the *Zernike*3*D* model, a volume *V*(**r**) can be moved towards a different conformation *V*′(**r**) through the application of a deformation field, 

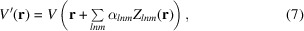

where *Z*_*lnm*_ are the *Zernike*3*D* basis functions and α_*lnm*_ are their coefficients.

For NMA, we must approximate *V*(**r**) by a set of pseudoatoms or atoms 

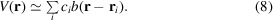

The deformation is performed at the level of NMA modes **u**_*k*_, 

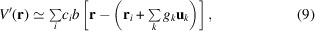

with coefficients *g*_*k*_.

The deformation according to NMA at point **r**_*i*_ must be equal to the deformation at the same point according to *Zernike*3*D*,

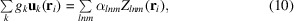

where **u**_*k*_(**r**_*i*_) is the restriction of the normal mode to the *i*th atom. We may write this as a system of linear equations, 

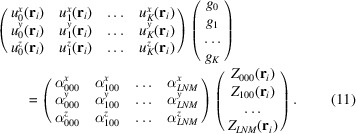

We may simplify the notation by introducing some matrices and vectors 

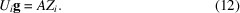

At this point, we simply need to consider all atoms from *i* = 1 to *i* = *P* by stacking them one below the other, 

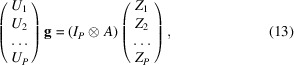

where *I*_*P*_ is the identity matrix of size *P* and ⊗ is the Kronecker product. This expression is very convenient for solving for **g** in a least-squares sense. The above equation is of the form 

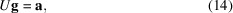

the least-squares solution of which is 

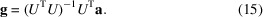

Equation (15) is the expression to convert the* Zernike*3*D* coefficients to normal mode coefficients.

*Conversion of NMA space to *Zernike*3*D* space*. Similarly to the previously presented case, it is also possible to define a closed expression for the backwards conversion. We start by transposing equation (12) to obtain

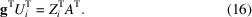

We now stack all of the information from all points,

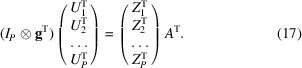

This system of equations is of the form 

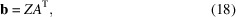

the least-squares solution of which is 

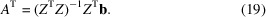

Equation (19) is the expression to convert a set of normal mode coefficients to *Zernike*3*D* coefficients.

### 2.2. Methods that directly estimate the deformed map

In this category, we give methods that do not estimate the deformation field **g** but directly estimate the deformed map *V*. We have so far integrated *cryoDRGN* into *Scipion*.

#### 2.2.1. cryoDRGN

*cryoDRGN* (Zhong *et al.*, 2021 ▸) is a new algorithm for the study of macromolecular flexibility based on a neural network specifically designed and trained to learn how to reconstruct cryoEM maps from a given set of particles. These particles should have a previously estimated rigid alignment and CTF information, which will be used by the network to learn how to decode the requested reconstructions.

The neural network design follows an autoencoder architecture. The encoder receives the input particles with alignment and CTF information, which are progressively downsampled towards a latent space of *N* dimensions specified by the user. Subsequently, the decoder transforms any point in the *N*-dimensional space into a full 3D reconstruction. By training on many particles, the network is also able to learn the specific conformational characteristics of the particles in the data set, which are captured by the latent space.

*cryoDRGN* has proven to be able to deal with both compositional and continuous heterogeneity at the cryoEM map level. *Scipion* Flexibility Hub integrates the main *cryoDRGN* pipeline, subdivided into a particle-preparation step and a network-training step. In addition, the output of the trained neural network has been connected to the tools implemented in the *Flexutils* plugin, allowing the user to interactively inspect and annotate *cryoDRGN* conformational landscapes in real time.

Similarly to the *Zernike*3*D* plugins, all of the information registered inside *Scipion* (such as the decoded volumes or the selected particles from the landscape) can be further refined with the SPA plugins integrated in *Scipion* [such as *RELION* (Scheres, 2012 ▸) or *cryoSPARC* (Punjani *et al.*, 2017 ▸)].

The *cryoDRGN* workflow is divided into two different protocols in the Flexibility Hub. The form associated with the previous protocols is shown in Supplementary Fig. S4.

The first step is called ‘Preprocess’ (Supplementary Fig. S4*a* ) and has been designed to wrap the preparation of the data needed by the *cryoDRGN* neural network. Input particles are the only mandatory input, which must have angular and CTF information. The output of this protocol will be registered as a set of *cryoDRGN* particles.

The second step wraps the training of the *cryoDRGN* network (Supplementary Fig. S4*b* ). Similarly to the pre­processing step, input particles are the only mandatory input. However, in this case it is necessary to provide a set of particles generated by the previous Preprocess protocol. The form includes different sections to modify the network architecture, as well as a way to provide any command-line argument that is not explicitly present in the form.

The protocol will register a set of particles extended using the *cryoDRGN* latent space vectors associated with the particles in the data set, as well as the information needed to reload the trained network.

### 2.3. Interactive annotation of conformational landscapes

Due to the intrinsic complexity of conformational landscapes, we have developed three interactive visualization tools in *Scipion* for the real-time inspection and annotation of conformational landscapes. The current implementation of the tools allows them to easily connect to the output of different algorithms, giving them a friendly and common interface where users can inspect the flexibility results that are obtained. Currently, the viewers have been connected to the *Zernike*3*D* (Herreros *et al.*, 2021 ▸) and *cryoDRGN* (Zhong *et al.*, 2021 ▸) outputs, allowing users to annotate any landscape estimated using these algorithms.

**Figure 5:**
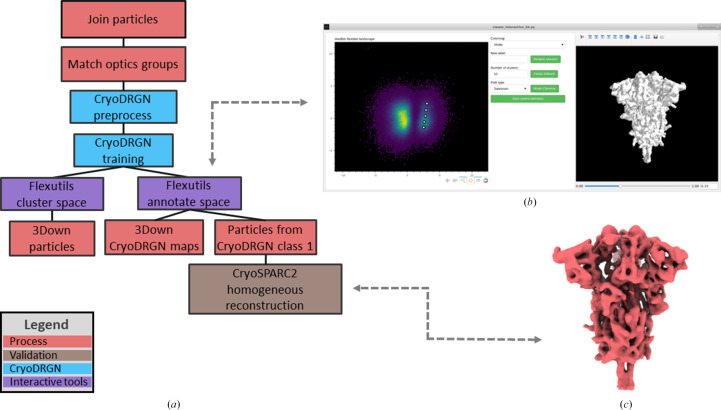
The *cryoDRGN* (Zhong *et al.*, 2021 ▸) Flexibility Hub workflow to analyse the continuous flexibility of the SARS-CoV-2 spike in the 1Up and 3Down conformations. (*a*) shows the current integration of the *cryoDRGN* pipeline in the Flexibility Hub, which includes two main steps: particle preparation and neural network training. The resulting conformational landscapes captured by the latent space of the trained network can subsequently be inspected and annotated with the *Flexutils* interactive tools. (*b*) provides an example of the 2D interactive annotation tool integrated into *Flexutils*. The viewer shows a hexbin plot resulting from a PCA reduction of the original latent space computed by *cryoDRGN*. Two main groups can be identified in this view corresponding to the 1Up and 3Down conformations of the spike. In addition, five points were selected along the main variability axis of the 3Down point cloud region. (*c*) shows an example of a *cryoSPARC* (Punjani *et al.*, 2017 ▸) homogeneous refinement with the closest particles associated with one of the five picked points (middle point) illustrated in (*b*). For this test, the 5000 original particles closest to the picked point were registered in *Scipion*, which led to a reconstructed volume with a resolution of 4.1 Å.

The viewers provide a low-dimensional representation (in 3D or 2D) of a conformational space estimated by PCA (Jolliffe & Cadima, 2016 ▸) or UMAP (McInnes *et al.*, 2018 ▸), which can be interactively annotated based on the real-time representation of the selected conformations, thus simplifying the selection and understanding of interesting conformational states. An example of the tools is provided in Figs. 5 ▸(*b*) and 6 ▸(*b*). The main difference between the 2D and 3D implementations of the tool is the information provided by the visualization: while the 3D version captures more features of the landscape thanks to its extra dimension, the 2D version provides more information on the internal distribution of the particles in the heterogeneity landscape.

The annotated space is registered automatically in *Scipion*, providing both maps and particles that can be used to obtain new conformations at high resolution using any standard processing software such as *cryoSPARC* (Punjani *et al.*, 2017 ▸) or *RELION* (Scheres, 2012 ▸).

Additionally, there are protocols to synthesize a given conformation based on direct application of the tool used to compute the landscape. For example, it is possible to apply the selected *Zernike*3*D* deformation coefficients to maps or structural models to yield the conformation of interest without the need to execute any refinement or reconstruction.

### 2.4. Additional analysis tools provided in the *Flexutils* plugin

Apart from the interactive annotation tools described previously, *Flexutils* integrates additional tools for the analysis of flexibility data. A brief description of the characteristics and usage of these tools is provided below.

(i) Motion statistics. One of the main advantages of algorithms designed to estimate deformation fields is the possibility of extracting local flexibility information from this representation. For example, it would be possible to estimate the average direction for every voxel or atom, as well as to determine which areas tend to deviate more from the original reference position. *Flexutils* provides a tool to compute such statistics from the deformation fields and visually represent the estimated information to simplify the analysis of the local characteristics of molecular motions. An example of this tool is provided in Fig. 2 ▸.
(ii) Best-view selection. Having a well distributed angular assignment in a population of particles is essential to achieve the desired processing results. However, particle views may contribute differently to the understanding of a given conformational change. Therefore, *Flexutils* provides an interactive tool to filter a set of particles according to the information that a given view provides about a specific conformational change described by a set of discrete cryoEM maps. In this way, it is possible to restrict the estimation of conformational landscapes to only the particle views that best explain the conformational changes of interest. An example of this tool is provided in Fig. 7 ▸. The tool relies on *ImageJ* (Schneider *et al.*, 2012 ▸) to perform the selection of the desired rotation–tilt angle combination giving rise to a particle subset that discretizes the most several conformational states.
(iii) Distance-based clustering of structural models. Apart from the automatic clustering based on *k*-means implemented in the interactive annotation tools, *Flexutils* includes a semiautomatic clustering approach in the atomic structure space. The structure space is currently generated through the application of deformation fields to a reference atomic structure, leading to a complete representation of the conformational changes of the particle at the atomic level. In addition, the user needs to provide a cutoff distance to determine the minimum root-mean-square distance (r.m.s.d.) among different clusters. The algorithm will progressively build as many clusters as possible from the atomic structure space that fulfil the cutoff distance condition, providing both the particles that have contributed to each cluster and the representative average structure computed during the clustering process.

## 3. Results

### 3.1. Combined Flexibility Hub workflow

One of the main advantages of the *Scipion* Flexibility Hub is the possibility of combining different flexibility algorithms to improve the quality of the information extracted from a given data set. The current integration allows one to seamlessly interoperate with different software, simplifying the design and execution of the flexibility workflow.

In order to illustrate the potential of combining several flexibility algorithms into a single workflow, this section provides a detailed explanation of the different tools and steps followed to analyse the continuous heterogeneity observed in a D614G SARS-CoV-2 spike data set acquired in the laboratory of Professor Subramaniam through the application of different flexibility algorithms.

The sample was prepared, collected and analysed using similar approaches to those reported for other SARS-CoV-2 spike data sets from the same laboratory (Mannar *et al.*, 2021 ▸), resulting in a set of 350 000 particles with a box size of 224 pixels and a sampling rate of 1.25 Å per pixel. The alignment of the particles and the contrast transfer function (CTF) information was estimated with *RELION* (Scheres, 2012 ▸), as this information should already be available to execute most flexibility algorithms. In addition, a 3D classification was carried out to confirm that the particles in the data set described the two main conformational states of the SARS-CoV-2 spike receptor-binding domains (RBDs): the 3Down and 1Up conformational states.

For simplicity, all of the preprocessing described above is excluded from the workflow to focus as much as possible on the presented results on the flexibility analysis of the spike.

The proposed workflow consists of a combined heterogeneity analysis of the spike using several algorithms including *cryoDRGN*, *Zernike*3*D* and *ProDy*. The orchestration of the algorithms relies on the *Flexutils* plugin, which is the core of the Flexibility Hub.

*Flexutils* integrates several tools to combine, analyse and process heterogeneity information. As an example of the capabilities of some of these tools, inspection and understanding of the spike conformational landscape were carried out using the interactive annotation interface provided by *Flexutils*, which is shown in Figs. 5 ▸ and 6 ▸. The interactive visualization allows the selection of any conformation in the landscapes, providing a real-time generation of the state associated with the current selection. In addition, it offers a shortcut to open any selected state in *ChimeraX* (Pettersen *et al.*, 2021 ▸) to further analyse the conformational changes without exiting the viewer.

Sections 2.3 and 2.4 provide a more detailed description of the abovementioned interactive visualization tools, as well as the other programs integrated into *Flexutils*.

#### 3.1.1. *cryoDRGN* analysis of the 3Down and 1Up spike conformational landscape

The first step performed in the flexibility workflow was to process the particle data set with *cryoDRGN* (Zhong *et al.*, 2021 ▸) as presented in Fig. 5 ▸(*a*). Currently, the integration of *cryoDRGN* in the Flexibility Hub framework is subdivided into two different steps: an initial particle-preparation step and a neural network training step. In the preparation step, the particles were downsampled to a box size of 128 pixels and a sampling rate of 2.19 Å per pixel. The preprocessed particles were then input into the training step in order to estimate the latent space representation of the conformational landscape from them.

The latent space from the *cryoDRGN* network can be inspected using any of the tools developed and integrated into the *Flexutils* plugin. An example of these tools is the 2D interactive annotation provided in Fig. 5 ▸(*b*). For this case, the dimensionality of the original *cryoDRGN* space was reduced to two dimensions by principal component analysis (PCA; Jolliffe & Cadima, 2016 ▸) and represented as a hexbin plot. Thanks to this representation, it is possible to easily identify the 3Down and 1Up clusters separated along the horizontal axis (PC1), confirming the results of 3D classification. *Flexutils* also has a non-interactive clustering protocol that allows us to extract larger sets of particles corresponding to main clusters in the data set such as all of the 3Down particles, as also shown in Fig. 5 ▸(*a*).

Compared with the tools provided by *cryoDRGN* for the analysis of its latent space, the tools implemented in *Flexutils* provide extra functionalities to simplify the inspection of conformational space. Firstly, the *Flexutils* tools overcome the need for any coding step by the user, making the inspection completely interactive. Moreover, the tools provide a real-time view of any selected conformation, as shown in Fig. 5 ▸(*b*), as well as a direct connection to *ChimeraX* (Pettersen *et al.*, 2021 ▸) for advanced tools. Lastly, the viewers implement 2D and 3D interactive visualization of the conformational landscape point clouds.

In addition, the tool allows the user to navigate between the points in the landscape, providing a real-time view of the conformation corresponding to any selected point. If several conformations are selected, it is also possible to open *ChimeraX* from the tool to view a real-time morphing of the different maps recovered from the network. In this way, it is possible to assess the different conformational changes captured by the latent space directly from initial inspection of the landscape.

In order to further analyse the flexibility of the spike, we selected five different conformations along the main variability axis of the 3Down cluster (the vertical axis in the plot; PC2), as illustrated by the white dots in Fig. 5 ▸(*b*). Thanks to this selection, *Flexutils* can register both the decoded maps recovered from the network and the closest particles surrounding each of the selected points in the landscape. In this way, it is possible to isolate the 3Down maps and particles, which can be used later in the workflow to connect to other flexibility tools.

Once the volumes and particles have been registered in *Scipion*, it is possible to process them using any SPA plugin integrated in *Scipion*. As an example, in Fig. 5 ▸(*c*) we provide a *cryoSPARC* (Punjani *et al.*, 2017 ▸) homogeneous refinement with the 5000 closest particles surrounding one of the conformations selected in the landscape. The possibility of refining selected conformations from the particles directly allows the recovery of high-resolution information about the selected structure, as the network does not learn this information due to the downsampling applied to the original particles during the preprocessing step.

#### 3.1.2. *Zernike*3*D* analysis of the conformational landscape of the 3Down spike

The* cryoDRGN* particles and maps previously extracted from the original particle data set can be used to connect the *cryoDRGN* workflow to the *Zernike*3*D* pipeline integrated into the Flexibility Hub. In this way, it is possible to take advantage of the versatility of *Zernike*3*D* to further analyse the continuous heterogeneity of the 3Down conformation at the atomic level, and to obtain extra information about the conformational changes exhibited by the spike through estimation of the *Zernike*3*D* deformation fields.

**Figure 6:**
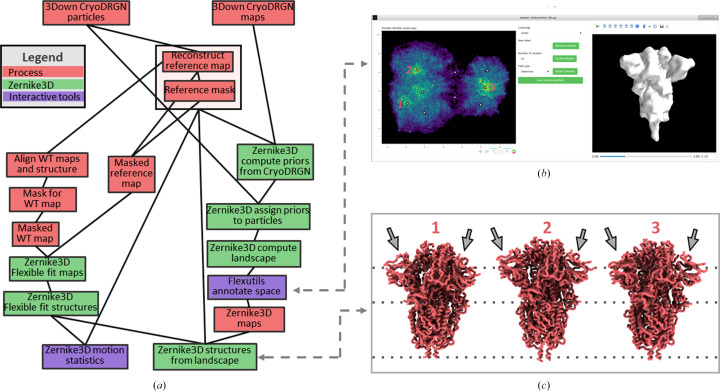
Example of the *Zernike*3*D* Flexibility Hub workflow connected to *cryoDRGN* to perform an advanced analysis of the variability of SARS-CoV-2 spike in the 3Down conformation. (*a*) shows the main pipeline followed to perform the *Zernike*3*D* analysis. The connection to the *cryoDRGN* results described in Fig. 5 ▸ is performed at both particle and volume levels to increase the reliability and compatibility of the landscapes. In addition, the workflow shows how to perform a structure flexible fitting with the *Zernike*3*D* protocols, which can be used to analyse the conformational changes in the landscape at the atomic level. (*b*) provides a UMAP representation of the estimated *Zernike*3*D* conformational landscape for the 3Down conformation. Several conformations were selected from the landscape based on the particle density information provided by the hexbin plot; they are marked with small white circles, and labels mark those points used in (*c*). (*c*) shows the structural changes retrieved at the atomic level thanks to the flexible fitting procedure presented in (*a*). The different structures correspond to the three labelled conformations selected in (*b*).

**Figure 7:**
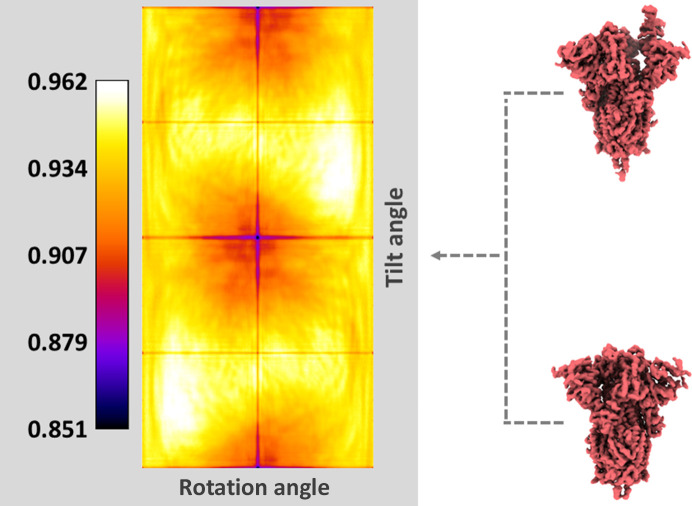
Example of the interactive selection of particle views providing more information on the 3Down to 1Up conformational change of the spike. From the original 3D conformations, a rotation–tilt heatmap is computed determining how good each combination of angles is at distinguishing between the two conformations analysed. In the heatmap, red colours correspond to the regions where the 1Up and 3Down conformations are better discriminated (*i.e.* the simulated projections differ the most). The color map represents the Pearson correlation values estimated from the simulated projections.

The *Zernike*3*D* workflow and its connection to *cryoDRGN* are provided in Fig. 6 ▸(*a*). The isolated 3Down particles and maps saved from the *cryoDRGN* landscape can be processed to generate the input data needed by the *Zernike*3*D* analysis, including a reference volume and a mask to focus the flexibility analysis on the protein. At this point, it is possible to mix all these pieces of information (particles and maps) in the same *Zernike*3*D* landscape to make the *cryoDRGN* and *Zernike*3*D* landscapes compatible with each other, as shown in the main branch on the right side of the workflow. This is achieved by initializing the *Zernike*3*D* landscapes following a multi-reference approach, instead of working with a single reference (the standard mode). Therefore, by initializing with some decoded *cryoDRGN* maps (five in this case) distributed across the conformational landscape, it is possible to converge towards landscapes that could be compared more easily.

Since the *Zernike*3*D* approach can work with maps, particles or structural models indistinguishably, the left branch of the workflow also includes a flexible fitting of a 3Down structure into the reference map required by the *Zernike*3*D* algorithm. The flexible fitting also relies on the *Zernike*3*D* tools, although it is also possible to use any of the other tools implemented in *Scipion* to obtain the fitted structure, such as the normal mode and molecular-dynamics method *NMMD* (Vuillemot *et al.*, 2022 ▸) in *ContinuousFlex*. Thanks to the flexible fitting, it is also possible to inspect the *Zernike*3*D* space at the atomic level to improve the representation of the spike motions and states.

Fig. 6 ▸(*b*) shows an example of the *Zernike*3*D* space reduced with UMAP (McInnes *et al.*, 2018 ▸) and analysed with the 2D annotation tools from *Flexutils*. The 3Down landscape is divided into two main regions, which can be inspected with the real-time annotation capabilities of the viewer. A total of nine conformations were selected in order to cover most of the interesting regions that can be observed in the representation. Similarly to the application of the tool to *cryoDRGN*, both the maps and the closest particles associated with the selections are registered inside *Scipion* to further process them if needed.

Lastly, the nine conformations that were extracted from the *Zernike*3*D* conformational landscape were synthesized at the atomic level thanks to the previously fitted structure. An example of three of these conformations is shown in Fig. 6 ▸(*c*). Thus, thanks to the combination of the *cryoDRGN* and *Zernike*3*D* tools, it is possible to more easily identify both global and local conformational changes of the spike (see Supplementary Video S3) and analyse them at different levels to provide further insights into the flexibility of the macromolecule under study (as shown in Fig. 2 ▸). One such motion is a separation of one of the N-terminal domains (NTDs) away from the core in conformation 1 compared with the others (see Fig. 6 ▸ *c*).

#### 3.1.3. NMA and atomic ensemble analysis of the 3Down spike landscape

**Figure 8:**
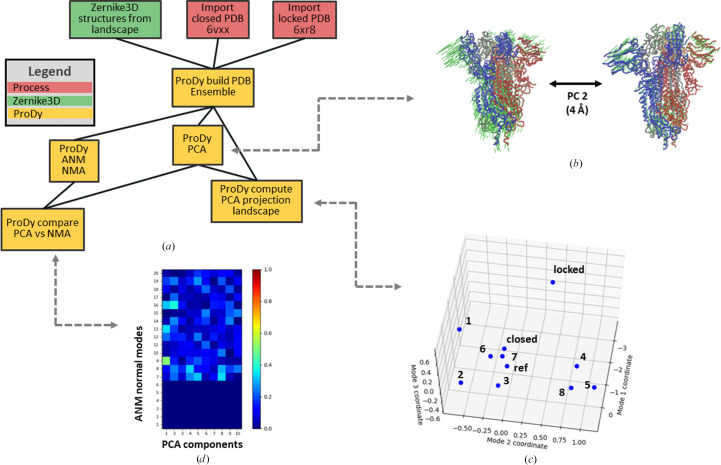
Example of the *ProDy* Flexibility Hub workflow connected to *Zernike*3*D* to perform an advanced analysis of the motions of the SARS-CoV-2 spike in the 3Down conformation. (*a*) shows the main pipeline followed to execute the *ProDy* analysis. The connection to the *Zernike*3*D* results described in Fig. 6 ▸ is performed using structural models in PCA and NMA. These structures as well as the canonical closed and locked structures (PDB entries 6vxx and 6xr8) are aligned into an ensemble that is then used for PCA, revealing motions such as that shown in (*b*). The conformational landscape can be visualized by a projection of the ensemble of aligned structures onto the first three principal components (PCs) as shown in (*c*). (*b*) shows an example of the motions along PC2, which contributes most of the variation (after PC1 that distinguishes the locked and unlocked conformations), using structures generated by adding the PC2 vector to the average structure using a vector size equivalent to an r.m.s.d. of 4 Å. (*c*) shows the *ProDy* PCA projection representation of the conformational landscape for the 3Down conformations selected from the *Zernike*3*D* landscape and the locked and closed conformations. It should be noted that the projections are presented on the scale of r.m.s.d.s, demonstrating a very small conformational variability of <1 Å for PC2 and PC3. (*d*) shows a comparison of the PCA components with the NMA modes from the aligned reference structure as a directional overlap matrix of correlation cosines coloured from low correlations in blue to high correlations in red.

The motions associated with the 3Down spike landscape can be assessed by PCA of the full ensemble of structures from the *Zernike*3*D* workflow or by NMA of any individual one of these structures using *ProDy* (Zhang, Krieger, Zhang *et al.*, 2021 ▸) or *ContinuousFlex* (Harastani, Vuillemot *et al.*, 2022 ▸). The calculated modes of motion can then be used in *ContinuousFlex* pipelines such as *HEMNMA* (Harastani *et al.*, 2020 ▸). We used *ProDy* for these steps as shown in Fig. 8 ▸(*a*), taking advantage of its richer toolkit for sequence-based and structure-based alignments.

The first step was to align the nine conformations from the *Zernike*3*D* analysis into an ensemble, reduce it down to a selection of C^α^ atoms and apply the *ProDy* iterative superposition procedure. We also included known experimental structures from the Protein Data Bank (PDB) in the canonical closed state (PDB entry 6vxx; Walls *et al.*, 2020 ▸) and the locked closed state (PDB entry 6xr8); Cai *et al.*, 2020 ▸) to compare our structural variation with the reported conformational change within the closed state.

This ensemble was then analysed by PCA, yielding three principal components (PCs) with fractional variances of 0.64, 0.17 and 0.07. Projection of the ensemble onto these PCs revealed a similar distribution to the that from *Zernike*3*D* in Fig. 6 ▸(*b*) (shown in Fig. 8 ▸ *c*), while providing interpretability of the motions underlying the landscape through the animation of the associated PCs using the *ProDy* normal mode wizard *NMWiz* (Bakan *et al.*, 2014 ▸) in *VMD* (Humphrey *et al.*, 1996 ▸).

PC1 separated the locked state from the others and clearly showed a tightening of all of the RBDs and NTDs (see Supplementary Video S4). PC2 exhibited an opening and closing of the NTDs relative to the RBD core, with one of the NTDs being dominant, as shown in Supplementary Video S5 and Fig. 8 ▸(*b*) (see also Fig. 6 ▸ *c* and Supplementary Video S3). PC3 exhibited a subtle reconfiguration of the RBDs and NTDs together with a small compaction and expansion of the membrane-proximal C-terminal helices (Supplementary Video S6), further distinguishing structures 1, 2 and 3.

To compare the principal components from the ensemble starting from the structural models, NMA was performed for the aligned flexibly fitted C^α^-atom reference structure from the ensemble using the anisotropic network model (ANM; Atilgan *et al.*, 2001 ▸; Eyal *et al.*, 2006 ▸) with the usual cutoff distance of 15 Å.

The correlation cosine overlaps between the resulting set of normal modes (NMs) and the set of principal components (PCs) were then calculated using the mode-comparison protocol, revealing significant overlaps of several pairs in the overlap matrix in Fig. 8 ▸(*d*). Unsurprisingly, this analysis picked up the first two nonzero normal modes as the most important: NM7 and NM8 show moderate correlation (0.30–0.35) with PC5 and PC8 and with PC4, respectively. Furthermore, it identified several other notable correlations, the strongest of which was NM9 with PC1 (0.51), followed by NM13 and NM16 with PC1 and PC2. This type of analysis could therefore help in the selection of appropriate modes for downstream analyses such as *HEMNMA*.

## 4. Conclusions

The ability to capture many conformational states at the particle level is pushing a paradigm change in cryoEM SPA. Thanks to the development of advanced algorithms designed to handle molecular flexibility, it is possible to estimate a richer conformational landscape from the particle heterogeneity information, leading to a better understanding of the heterogeneity of the specimen under study.

There is, however, a lack of consensus in the information handled by these algorithms, increasing the difficulty of combining them into a single flexibility workflow. Nevertheless, it is only through the combination of many algorithms that is possible to take advantage of their strengths, allowing users to extract more accurate information to study the molecular motions that a molecule may undergo.

Therefore, this work introduces a new integrative framework in *Scipion* (Rosa-Trevín *et al.*, 2016 ▸) called the Flexibility Hub, the aim is of which to simplify the interoperability of novel and popular heterogeneity algorithms. In this way, it is possible to easily transfer the results from one piece of software to another without the need for any user intervention in the conversion process, helping in the design of more complete flexibility workflows. Currently, two main approaches to estimate conformational variability at different levels are available in the framework: software estimating deformation fields to describe motions (*Zernike*3*D*, *ProDy* and *ContinuousFlex*) and algorithms that are trained to reconstruct heterogeneous maps from cryoEM particles (*cryoDRGN*).

In addition, the Flexibility Hub integrates several analysis tools that are compatible with the different software supported and pluginized inside *Scipion*, such as interactive annotation and landscape-exploration tools or the automatic registration of particles and heterogeneous reconstructions obtained from the estimated conformational landscapes.

As a guide to the current ‘road map’ for Flexibility Hub, we indicate that future steps will include the integration of new heterogeneity software such as 3*DFlex* (Punjani & Fleet, 2022 ▸), Gaussian mixtures models (Chen & Ludtke, 2021 ▸) and *ManiFoldEM* (Frank & Ourmazd, 2016 ▸), allowing them to interface with the previously integrated packages and tools. Effort will also be focused on the development of advanced consensus and comparison tools that are able to compare and generate more reliable conformational landscapes.

Apart from integrating new software into the Flexibility Hub, we will implement future tools to provide an automatically generated summary report explaining the conformational landscapes and possible conformational changes that are present in the data set. The idea of such a report is to give users some insights into the heterogeneity of their data, allowing them to more easily understand the information that is contained in the estimated landscapes.

Moreover, there will be a direct interface with *ScipionTomo* (Jiménez de la Morena *et al.*, 2022 ▸) to extend the functionalities and heterogeneity workflows to cryoET.

## 5. Code availability

All of the algorithms described here are available in the following *Scipion* plugins: *Zernike*3*D*, *scipion-em-xmipp* and *scipion-em-flexutils*; *ContinuousFlex*, *scipion-em-continuousflex*; *ProDy*, *scipion-em-prody*; *cryoDRGN*, *scipion-em-cryodrgn*. 

## Supplementary Material

Supplementary Video S1. Comparison of conformational states before and after focusing the ribosome conformational landscape on the L1 stalk region as shown in Fig. 3. DOI: 10.1107/S2059798323004497/ic5121sup1.mp4

Supplementary Video S2. Morphing of the two conformational states displayed in Fig. 4 reconstructed with ZART with reference reassignation. DOI: 10.1107/S2059798323004497/ic5121sup2.mp4

Supplementary Video S3. Movie showing the morphing of several spike conformations extracted from the Zernike3D conformational landscape shown in Fig. 6(b). DOI: 10.1107/S2059798323004497/ic5121sup3.mp4

Supplementary Video S4. Movie showing motions along PC1 scaled to an r.m.s.d. of 4 Å. DOI: 10.1107/S2059798323004497/ic5121sup4.mpg

Supplementary Video S5. Movie showing motions along PC2 scaled to an r.m.s.d. of 3 Å. DOI: 10.1107/S2059798323004497/ic5121sup5.mpg

Supplementary Video S6. Movie showing motions along PC3 scaled to an r.m.s.d. of 1.5 Å. DOI: 10.1107/S2059798323004497/ic5121sup6.mpg

Supplementary Figures. DOI: 10.1107/S2059798323004497/ic5121sup7.pdf
