# Supplementary material for: Scipion Flexibility Hub: an integrative framework for advanced analysis of conformational heterogeneity in cryoEM
Source: Acta Crystallogr D Struct Biol. 2023 Jun 16;79(Pt 7):569–84. doi: 10.1107/S2059798323004497 (PMC10306064; doi:10.1107/S2059798323004497)
Supplement: Supplementary file 7 [file d-79-00569-sup7.pdf]

STRUCTURAL
BIOLOGY

Volume 79 (2023)

Supporting information for article:

Scipion Flexibility Hub: an integrative framework for advanced analysis of conformational heterogeneity in cryoEM

D. Herreros, J. Krieger, Y. Fonseca, P. Conesa, M. Harastani, R. Vuillemot, I. Hamitouche, R. Serrano Gutiérrez, M. Gragera, R. Melero, S. Jonic, J. M. Carazo and C. O. S. Sorzano

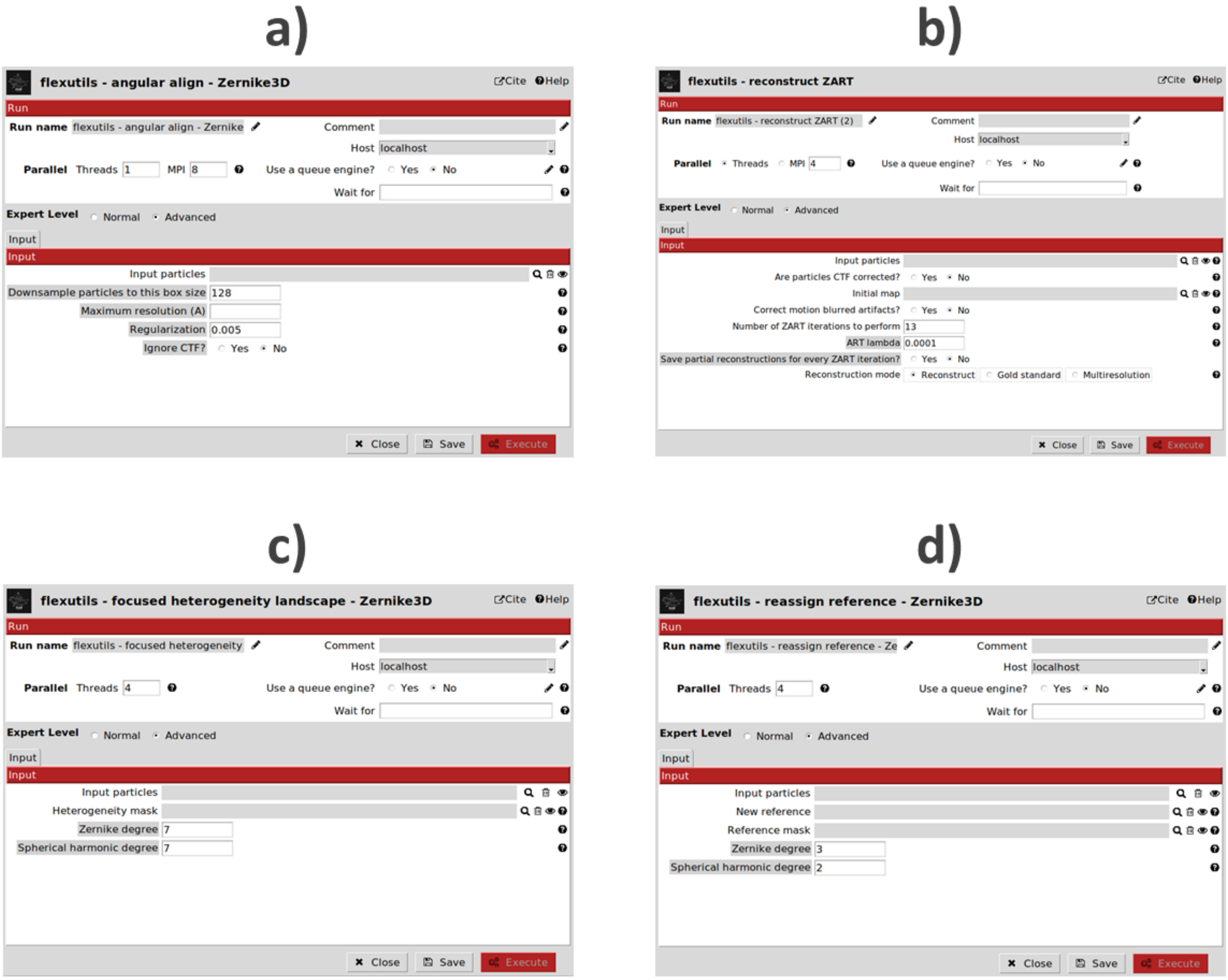

Suplemmentary Fig. S1: Example of input forms for some Zernike3D protocols. a) shows the input form of the Zernike3D coefficient estimation program. Depending on the information stored in the input particles, other parameters might be offered dynamically. b) shows the input form of ZART protocol. The only mandatory input is the set of particles to be reconstructed with angular assignation and CTF (unless particles have been CTF corrected). Heterogeneity correction needs that particles have been generated by a Zernike3D coefficient estimation protocol. c) shows the form of the focused landscape protocol. In this case, the protocol needs both, the input particles (generated from a Zernike3D coefficient estimation run), and a mask determining the region where the landscape will be focused. d) shows the form of the reassign reference protocol. Similarly to the previous case, the protocol needs a set of particles with Zernike3D coefficients estimated, and two masks determining the area where the new and old references are located in the volume.

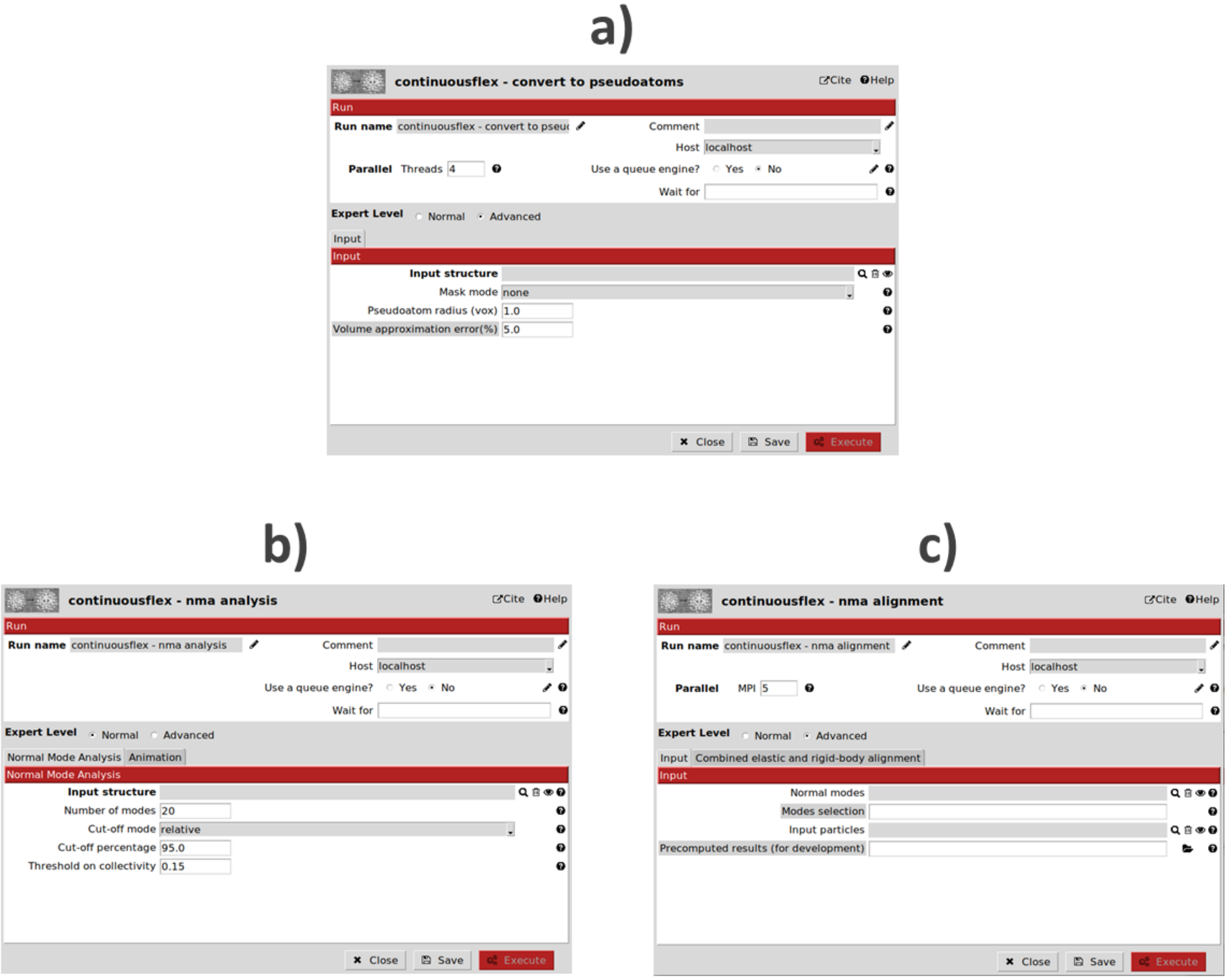

Suplemmentary Fig. S2: Example of input forms for some ContinuousFlex protocols. a) shows the input form of the volume to pseudoatoms conversion protocol. This program is useful when an atomic structure of the macromolecule being analyzed is not available, as it converts a given CryoEM map to a representation adequate to perform a Normal Mode Analysis (NMA). b) shows the input form of NMA analysis protocols. This protocol computes a series of normal modes from the input structure provided. The input structure could be a real structure or a pseudoatomic structure extracted from a map through the protocol shown in a). c) shows the form of the estimation of per-particle Normal Mode coefficients. Inputs required include the particles to be analyzed (that must have angular information and CTF assigned), and a set of normal modes estimated with the protocol described in b).

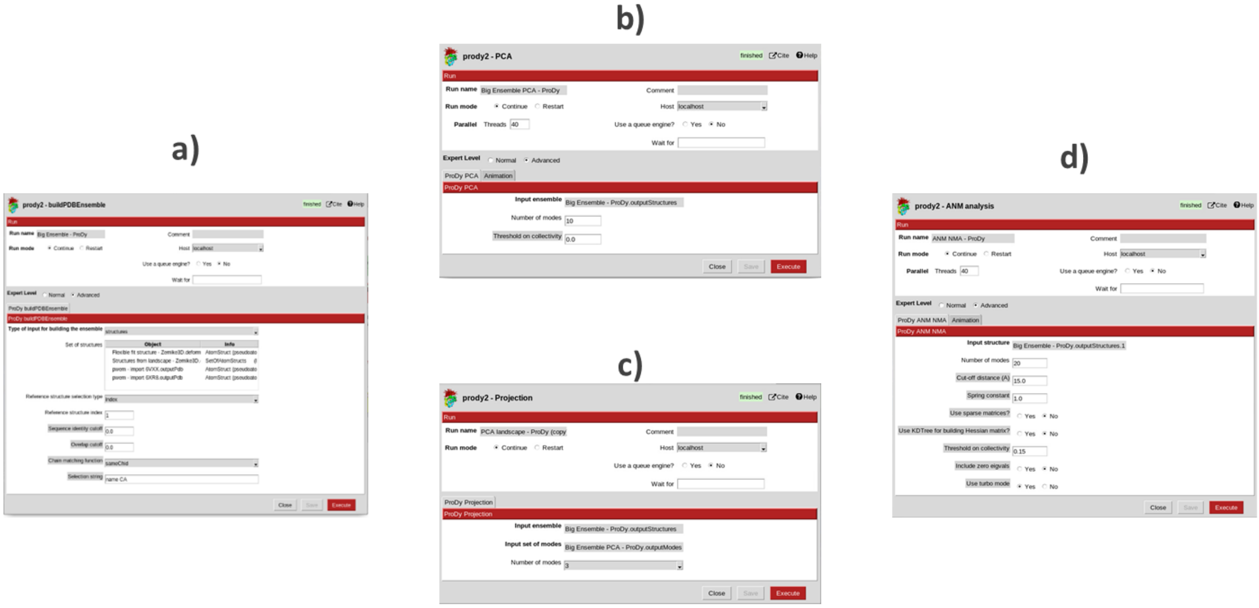

Supplementary Fig. S3: Example of input forms from some ProDy protocols. a) shows the input form of the ensemble building step needed for PCA. Input conformations can come from isolated atomic structures or sets of atomic structures. b) shows the form of the PCA protocol. The ensemble passed to the form is that previously generated in the ensemble building step. c) shows the form of PCA projection protocol for generating landscapes. This protocol needs both the ensemble and the set of principal components (or a set of normal modes) as well as a number of components or modes (1, 2 or 3). d) shows the form of the anisotropic network model (ANM) NMA protocol. It takes a single structure as input and has advanced parameters for the ANM and NMA.

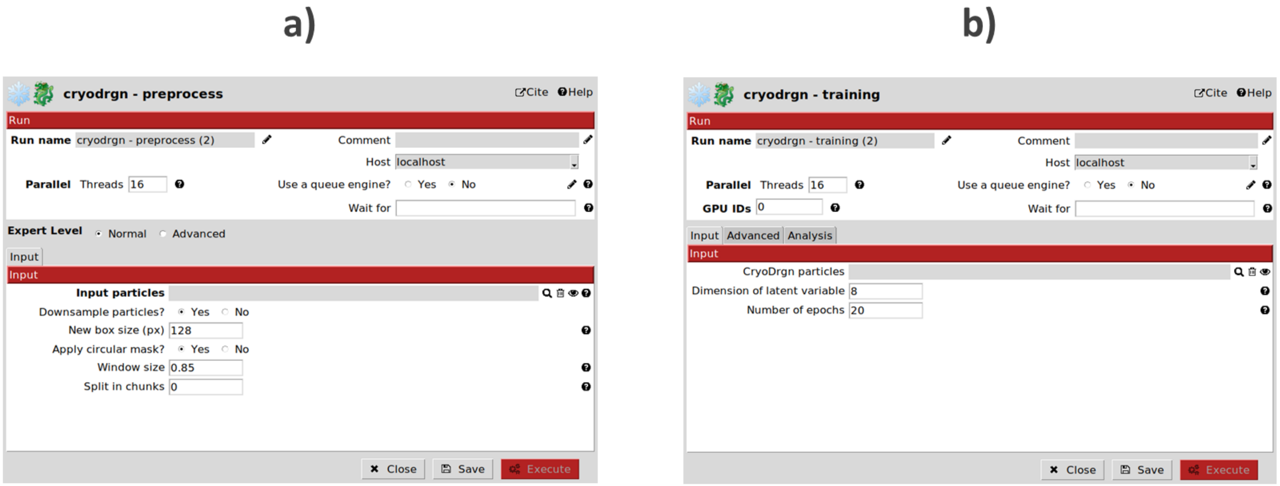

Suplemmentary Fig. S4: Example of input forms from some cryoDRGN protocols. a) shows the input form of the preprocessing step needed to generate the particles to be fed to the network. The input particles must have angular and CTF information. b) shows the form of the training protocol. The particles to be passed to the form are those previously generated in the preprocessing step. The parameters in the form are a simplification of the cryoDRGN command line inputs. However, it is still possible to pass any extra parameter not considered in the form through an input field found inside the "Advanced" section.
